# Developing Personal Resilience Questionnaire for rural doctors: an indigenous approach study in Indonesia

**DOI:** 10.1186/s40359-021-00666-8

**Published:** 2021-10-15

**Authors:** Nicholas Edwin Handoyo, Gandes Retno Rahayu, Mora Claramita, Marselino K. P. Abdi Keraf, Karol Octrisdey, Kwartarini Wahyu Yuniarti, Julie Ash, Lambert Schuwirth

**Affiliations:** 1 Faculty of Medicine, University of Nusa Cendana, Jl. Adi Sucipto, Penfui, Kupang, Nusa Tenggara Timur Indonesia; 2 Department of Medical, Health Professions Education, and Bioethics, Faculty of Medicine Nursing and Public Health, University of Gadjah Mada, Yogyakarta, Indonesia; 3 Faculty of Psychology, University of Nusa Cendana, Kupang, Nusa Tenggara Timur Indonesia; 4 Polytechnic of Health, Kupang, Nusa Tenggara Timur Indonesia; 5 Faculty of Psychology, University of Gadjah Mada, Yogyakarta, Indonesia; 6 Prideaux Discipline of Clinical Education, Flinders University, Adelaide, SA Australia

**Keywords:** Linguistic and cultural adaptation, Resilience, Questionnaire, Doctor, Rural, Indonesia

## Abstract

**Background:**

Resilience is recognized as a critical component of well-being and is an essential factor in coping with stress. There are issues of using a standardized resilience scale developed for one cultural population to be used in the different cultural populations. This study aimed to create a specific measurement scale for measuring doctors’ resilience levels in the rural Indonesian context.

**Method:**

A total of 527 rural doctors and health professional educators joined this study (37 and 490 participants in the pilot studies and the survey, respectively). An indigenous psychological approach was implemented in linguistic and cultural adaptation and validation of an existing instrument into the local Indonesian rural health context. A combined method of back-translation, committee approach, communication with the original author, and exploratory qualitative study in the local context was conducted. The indigenous psychological approach was implemented in exploring the local context and writing additional local items.

**Result:**

The final questionnaire consisted of six dimensions and 30 items with good internal consistency (Cronbach’s α ranged 0.809–0.960 for each dimension). Ten locally developed items were added to the final questionnaire as a result of the indigenous psychological approach.

**Conclusion:**

An indigenous psychological approach may enrich the linguistic and cultural adaptation and validation process of an existing scale.

**Supplementary Information:**

The online version contains supplementary material available at 10.1186/s40359-021-00666-8.

## Background

The concept of resilience is getting more attention, and it has been suggested that the training of health practitioners should support the development of resilience [1–7]. However, there are many different definitions [8, 9] and resilience measurements, yet none were considered ‘gold standard’ [10]. For this study, we use the concept of personal resilience in adults. Personal resilience is defined as the ability of a person to rebound, spring back, and have the flexibility or recuperability that enables one to thrive in the face of adversity [11, 12]. A palm tree curving in the wind and returning to its original positions illustrates resilience [2].

Resilience is acquired through an interaction between a person and environment in the form of stressors, adversities, opportunities, and other changes [1, 8]. Thus, the development of resilience is context-specific and influenced by geography and culture [13]. Thus, a measurement developed in other geographic and cultural contexts may not be valid to be used in different contexts [14, 15], and adaptation of such an instrument is always needed. For example, the 25 items of Wagnild and Young’s resilience scale had two-factor structures in a Norwegian sample [16]. The validation studies in Nigeria [17] and Portugal [18] yielded three and five-factor structures. For this, the mere translation of an instrument from one language to another is likely not to be sufficient for cross-cultural adaptation (CCA) [15, 17–19], and careful transculturation is required as well.

Indigenous psychology contends that psychological theories are not universal and advocate that a psychological construct, including resilience, be examined and understood in its social, religious, and cultural context. An indigenous approach suggests that researchers modify and adapt psychological theories and integrate them into the local cultural knowledge [20]. A cup of coffee with different tastes in various parts of the world illustrates an indigenous approach. Although the primary component is the same (coffee), it may be sweeter or bitterer to be better accepted by different populations. But it is still coffee, and it does not necessarily change into tea.

As part of a larger project to develop resilience and increase doctors’ retention in the rural areas of Indonesia, an instrument to assess their resilience was needed, and most instruments were developed for a different context. This paper, therefore, describes the process of linguistic and cultural adaptation and validation of the Four Dimensions Adult Personal Resilience Questionnaire [12] to the context of Indonesian rural doctors. However, this study is not just aimed at developing a questionnaire specifically measuring rural doctors’ resilience in the Indonesian context but also at learning more about validating a questionnaire for use in a different setting. The hypothesis of the study was the original questionnaire items works in the Indonesian context.

## Methods

### Context

This study was conducted in the Nusa Tenggara Timur (NTT) province, one of the Indonesian rural provinces. NTT is an archipelago province located at the border of Indonesia and consists of 1192 islands, among which only 432 islands have names and 44 inhabited [21]. The majority of its areas are considered underserved areas [18 out of 22 districts] [21, 22]. This widespread region of islands has a culturally diverse but predominantly Christian (90%) population of 5.28 million. The doctor-population ratio is 14 per 100,000, far lower than the recommended WHO ratio of 100/100.000 [23].

### Design

A cross cultural adaptation and validation study was conducted in May–November 2018. We combined the approaches suggested by Eun-Seok Cha and Julianne Callegaro Borsa, which includes: a back-translation method, bilingual technique, a committee approach, communication with the author of the original instrument, and a pre-test procedure [15, 18]. We added an indigenous psychological approach [20] to those combined approaches. Pre-test refers to a pilot study in a smaller sample of the population with similar characteristics and conditions to the actual research to identify potential problems in translation equivalence [18]. The last step was an online cross-sectional survey to validate the questionnaire in September–October 2018.

**Fig. 1:**
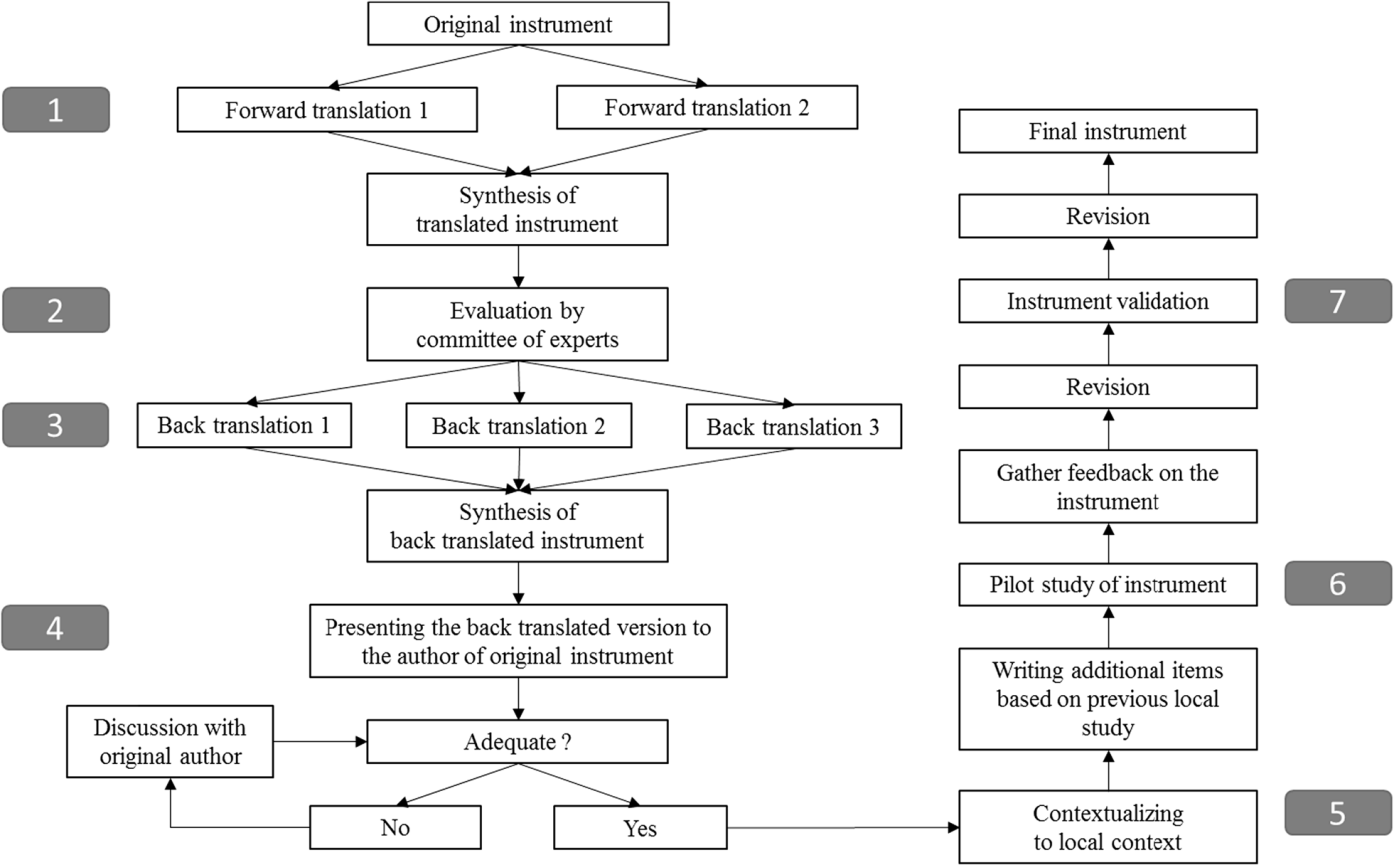
Study flow

In summary, the overall process involved seven steps (Fig. 1). These steps are simplified into three processes: translation (step 1–4), contextualization (step 5), and validation (step 6–7).

#### Step 1. The translation process

The translation process combined the forward and the backward translations, committee approach, and consulting the author of the original questionnaire [15, 18]. The committee approach refers to the use of a group of bilingual experts [18]. First, the initial questionnaire (English version) was translated by the first researcher (NH). Then NH sent both the English and the Indonesian version to the second translator for review (translator A). NH and translator A discussed the review result until reaching a consensus. The result was designated as Indonesian version 2, then sent for expert team evaluation (GR, MC, and KY). They evaluated the translated questionnaire by comparing each items’ meaning between the original and the Indonesian version 2, assessed its acceptance in Indonesian culture, and suggested some words modifications if needed to increase its acceptance.

The Indonesian version 2 was considered acceptable, and no change was made after the evaluation. NH then sent the Indonesian version 2 to be back-translated by three independent translators (translator B, C, and D). NH compared the three back-translated versions for every item, chose the most similar translation to the original English version, and then compiled it together to become the back-translated version. The comparison of items in its original, back-translated, and Indonesian version 2 was then structured in an excel file and presented to the author of the initial questionnaire [12], Prof T, for review. Despite some vocabulary differences between the original and back-translated versions, Prof T confirmed all equivalent items.

We intentionally chose translators from diverse backgrounds to obtain the broadest linguistic and target population perspectives [19]. The translators had different acculturation levels to English-speaking countries. The first translator was the first researcher (NH), an Indonesian male doctor working in NTT province and a Ph.D. candidate in Medical Education. Translator A was a female Indonesian-born registered nurse who works in a public hospital in New South Wales, Australia. She is married to an Australian and previously worked as an Australian certified interpreter and translator. Translator B was an Indonesian male with a degree in English literature who worked as an English professional translator in Indonesia. Translator C was an Indonesian male studying in an Australian university as a Ph.D. candidate in Psychology. Translator D was an Indonesian female doctor pursuing a Ph.D. in Public Health at an Australian University.

All step 1 process was conducted online. We used emails to connect people in five locations and three countries: Kupang (NH) and Yogyakarta (GR, MC, KY, and translator B) in Indonesia, Wolongong (translator A) and Adelaide (translator C and D) in Australia, and Macau in China (Prof T).

#### Step 2. The contextualization process

An approach called indigenization from without was implemented. An existing instrument was examined and modified to fit the local cultural context by exploring the related themes in the local cultural context [20]. NH conducted the process by comparing the APRQ’s items and dimensions to the codings identified in our previously undertaken exploratory qualitative study on motivations, characteristics, and factors related to the long-term retention of general practitioners in NTT Province, rural Indonesia [24]. In the previous study conducted in 2012, NH interviewed rural doctors working in NTT province for more than ten years. NH explored their motivations, characteristics, and factors related to their retention in rural areas. He found several internal (e.g., self-actualization, God’s call, rural attachment, etc.) and external (e.g., family, politic, etc.) factors related to their retention. NH and a psychologist (KY) compared the codings developed in the previous study to APRQ’s items and found that the APRQ items did not sufficiently cover several codes in the previous study. Thus, NH and KY wrote 30 related items based on the participants’ verbatim transcripts (Table 1). Literature suggested that researchers write at least 2–3 times more than the number of items needed [25]. NH added these 30 items to the 20 items translated from the original questionnaire to forming a 50-item Indonesian version.

**Table 1:** The local written items were based on the previous study codes

No	Local study code	Item
1	Social relationship	In my spare time, I enjoy spending time with my colleagues
2	Social relationship	I feel at home because my family lives here
3	Social relationship	I feel accepted by the community here
4	Feeling settle	Since I moved here, I often feel anxious at night time
5	Feeling settle	I feel financially secure
6	Feeling settle	I am satisfied with my career
7	Entrepreneurship	I feel that there are many opportunities here to advance my career
8	Rural attachment	I like living here
9	Rural attachment	I enjoy being able to assist those in the community who most need my help
10	Rural attachment	I have fond memories of this place
11	Rural attachment	This is the right place for me
12	Rural attachment	This place feels familiar to me as if I’ve lived here all my life
13	Rural attachment	I feel like part of the community here
14	Satisfaction	I am financially better off here than in other places
15	Satisfaction	Since I started working here, I have been able to achieve many of my goals (dreams)
16	Satisfaction	Since I started working here, I have been able to save money for the future
17	Satisfaction	I really enjoy living in this place
18	Satisfaction	Moving here for work was a good decision
19	Life calling	I believe that God has a purpose in placing me here
20	God guidance	God has always guided me through my life
21	God guidance	God would never allow me to walk through life alone
22	God guidance	Even in difficult situations, God has been there to guide me
23	God guidance	I believe that God is ever-present in everything I do
24	Life choice	My way of life is my choice
25	Responsibility	I always try to finish what I start
26	Responsibility	I feel obliged to contribute to the development of this community
27	Responsibility	I feel that I have a moral responsibility to this community
28	Self-actualization	I feel that my efforts as a doctor are appreciated
29	Self-actualization	I feel that my presence here is helping others
30	Self-actualization	The choice to remain here is not solely driven by financial income

Only the previous study’s codes not available in the original APRQ are presented

**Fig. 2:**
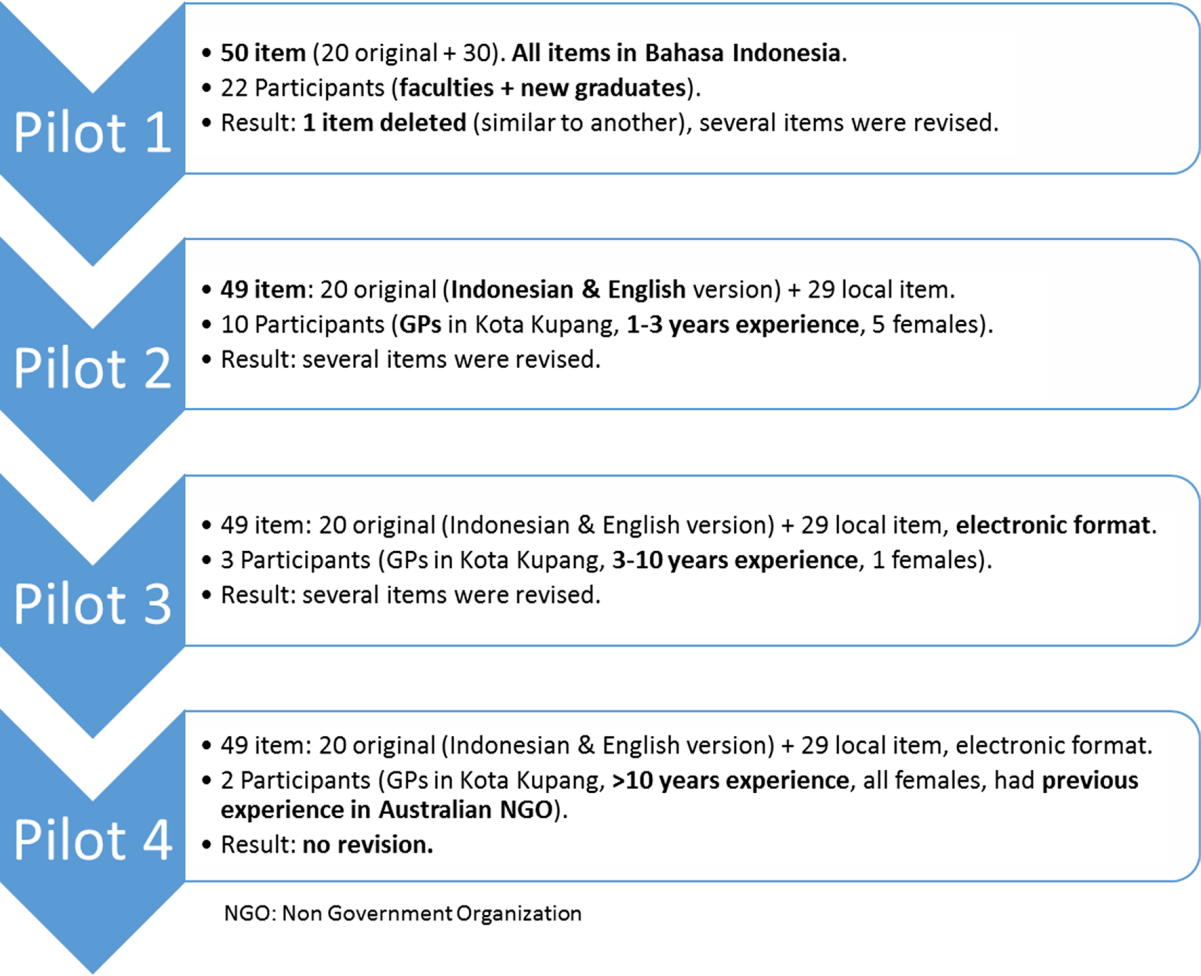
Pilot studies

NH facilitated the pre-test of the 50 items version in pilot studies on people with similar characteristics to the target participants (Fig. 2). The purpose was to collect feedback for instrument improvement, especially its appearance, the items’ wording, the perceived meaning of the items, technical difficulties in completing the online version, and additional comments about the instrument. This process is also called cognitive interviews [26].

NH questioned the participants using an interview guide (Table 2), especially on the participants’ response differences in the English and Indonesian item versions. NH conducted either group or individual face-to-face meetings for the first two pilots in the Nusa Cendana Medical School meeting room in Kupang. NH conducted phone interviews in the last two pilots, for which the participants filled out a fully electronic version of the questionnaire. All interviews were audio-recorded (only the first pilot was video-recorded). Only NH and the participant were present in the discussions, except for the group meeting, a co-facilitator helped with the logistics. NH performed all interviews only once. The duration of the interviews ranged between 5–24 min, depending on the number of different responses found between the Indonesian and English versions. Since it was an improvement on the individual items that were sought, data saturation was not necessary. The interviews were not transcribed, but NH asked participants to confirm their responses and suggestions at the end of each interview. NH wrote field notes and summaries after each discussion.

**Table 2:** The pilot studies interview guide

No	Question
1	What do you think about the questionnaires? Are there any difficulties you faced in filling out the questionnaire?
2	Do you have any suggestions on the appearance of the questionnaire?
3	Is the wording of the items easy to understand?
	Do you feel any difference in meaning between the Indonesian and English versions? Is that difference drive you to give a different response? Do you have suggestions to improve it?
4	Do you have any additional comments or suggestions?

NH deleted one item after the first pilot, as suggested by the participants. The 49 items version continue to the second pilot onward. Every revision made on the items starting from pilot study 1 was based on the participants’ inputs and consultations with a psychologist. The first researcher (NH) discussed each item’s meaning, its indicator, and the participants’ feedback with a local psychologist (AK) in a face-to-face meeting to agree on the final revised items. Every revision made was recorded in the Microsoft Word document. No modifications were made in the fourth pilot study. Therefore it was considered ready to be validated using a wider group of participants.

#### Step 3. The validation process

NH wrote the 49 items questionnaire with a 1–5 Likert scale in a google form. NH delivered the form electronically by email or WhatsApp to 737 registered doctors working in all NTT districts except Kota Kupang, the capital city, because the doctors in Kota Kupang had participated in the pilot studies 1–4 above. The WhatsApp social media application was chosen as an alternative to email since many people in NTT Province use this social media application and open it frequently. Reminders were sent to non-responders in a week and two weeks after the previous reminder [27].

The criteria of having ten times the number of participants (490) as items (49) were recommended by literature for conducting an optimal Exploratory Factor Analysis (EFA) [28, 29]. Another literature suggested having a minimum sample of 130 for 49 variables with six factors and wide communality [30]. The data consisted of 49 item variables score from 1 to 5. Each item was marked as a required field on the questionnaire to ensure no missing data.

NH and a statistician (KO) conducted the psychometric analysis on the first 490 participants’ responses to examine the descriptive statistic, construct validity, and reliability analysis. NH and KO reviewed the data normality using Shapiro–Wilk and the distribution plots resulting in a non-normal distribution. Non-normal distribution happened when the p value of Shapiro–Wilk < 0.05 [28]. NH and KO removed outliers based on all items sum score using boxplots, filtering data with ‘149 < sum scores < 227’, and re-examining the data normality. The result was still non-normally distributed data. NH and KO then examined the descriptive statistic, the construct validity, and the reliability for the remaining 454 participants.

*Descriptive statistic*. Sociodemographic variables were presented as frequencies and percentages for categorical variables and mean and standard deviation for numeric variables.

*Construct validity*. EFA with direct oblimin rotation was implemented to examine the construct validity of the questionnaire. EFA was chosen since this was a newly adapted questionnaire used for the first time in an Indonesian sample [26, 29]. An adjustment for the negative question was made before starting the EFA.

The EFA includes several steps. Firstly, choosing the approach. Instead of principal component analysis (PCA), a factor analysis approach was chosen because factor analysis was believed to estimate latent constructs that cannot be measured directly from the data [28, 31], such as resilience dimensions. Second, choosing principal axis factoring (PAF) as the extraction method. PAF is recommended to give the best result for non-normally distributed data [31]. Third, determining the number of factors to be retained. The decision on the number of factors to be extracted was made by taking into account the Kaiser’s criterion (eigenvalues > 1 rule), the total variance explained [28, 29, 32], the scree plot [28, 29, 31, 32], and parallel analysis [31, 33] that were available in the SPSS and JASP statistical programs that we used. The theoretical plausibilities and the total variance explained were considered in the judgment of the most appropriate factor solutions to be used among several alternatives [29].

Fourth, choosing the rotation method. Since those were the resilience dimensions being examined, it was assumed that the dimensions/factors were not independent. Therefore, direct oblimin rotation was chosen instead of varimax rotation to allow correlations between factors [28, 31, 32]. Fifth, we chose the scoring option for missing value as ‘exclude cases listwise.’ Sixth, the suitability of the sample for conducting EFA was examined using the Kaiser-Mayer-Olkin (KMO) measure of sampling adequacy and Bartlett’s Test of Sphericity [28, 29]. The Kaiser–Meyer–Olkin (KMO) score of 0.93 above the acceptable limit of 0.5 verified the sampling adequacy [28].

Seventh, the criteria of factor loading at least 0.30 [31] and the theoretical basis of the extracted factor were considered when deleting or retaining items [28, 29, 32]. Eighth, EFA was re-run and re-examined every time an item/s were deleted until we found a satisfying model. Finally, NH and KO conducted confirmatory factor analysis (CFA) to test the resulting model after the EFA.

*Reliability analysis*. Cronbach’s alpha was calculated for each dimension to examine the questionnaire’s internal consistency. Acceptable values range from 0.70 to 0.95 [28, 34].

We employed several strategies to ensure rigor in the process. We involved psychologists in the process, and we consulted the original questionnaire’s author to ensure the explored concept has the same meaning as the initial questionnaire [15]. Local people with diverse backgrounds (e.g., professions, sex, religions, hometown origins, and length of work experience) were involved in the process [19] as translators and participants to ensure the items were relevant, meaningful and acceptable in the local culture. A professional translator (translator B) and several other bilingual people were employed as translators, expert committee members, and pre-testing/pilot participants to ensure the items had the same meaning [18]. We implemented an indigenous psychological approach to ensure that the questionnaire reflecting the local cultural understanding of the construct and the local people’s behavior appropriately [20].

### Participants

A total of 527 participants joined this study. The participants can be divided into the pilot study groups (pilot study 1–4) and the survey group (Table 3).

**Table 3:** Distribution of participants

Step	Male	Female	Subtotal	Detail
Pilot 1	6	16	22	12 med school lecturers, 10 new graduates
Pilot 2	5	5	10	GPs 1–3 years of practice
Pilot 3	2	1	3	GPs 3–10 years of practice
Pilot 4	0	2	2	GPs > 10 years experience with previous experience in Australian NGO
Survey	202	288	490	GPs in NTT province (exclude Kupang City)
Total			527	

The pilot studies participants were selected purposively. The inclusion criteria were doctors who are also bilingual (able to read and speak in Bahasa Indonesia and English). But, an exception was made for the first pilot in which lecturers were involved, not all doctors. The various participants provided diverse perspectives on the questionnaire by including a view from people involved in teaching medical students. Involving medical teachers was related to the purpose of the larger project, as explained in the introduction section.

The first pilot participants were recruited at the end of the annual problem-based learning (PBL) tutor training workshop held by the Nusa Cendana Unversity, Faculty of Medicine. This training was attended by all lecturers and new graduates seeking a tutor position. The first researcher explained the research aim and what was required from the participants, asked for participation, delivered the paper-based questionnaire, which was completed and discussed.

The second to fourth pilot group participants were recruited purposively by considering their sex, institution, practice duration, and English ability. They were contacted by WhatsApp or SMS, informed about the research, and then asked to participate. All of the pilot study participants were colleagues of the first researcher (NH). Only one participant in pilot 3 failed to join because of her high workload.

For the fully developed post-pilot survey, group participants were GPs registered at either the Indonesian Doctors Association NTT branch or local district health offices. All of the registered GPs were contacted by WhatsApp or SMS, informed about the research, asked to participate, and provided a link that explained the study, informed consent, and the online version of the questionnaire.

### Instrument

The Four Dimensions Adult Personal Resilience Questionnaire (APRQ) developed by Taormina [10, 12] was chosen amongst many resilience measurements available in the literature. It was considered the most straightforward instrument that was validated in health workers, which was most appropriate and acceptable for the rural Indonesian context. It identifies resilience as consisting of four dimensions: determination, endurance, adaptability, and recuperability [9, 12]. The determination meant the firmness of purpose to succeed. Endurance meant the strength to withstand difficult situations faced without giving up. Adaptability meant the capacity to cope with adverse environments and adjust oneself to fit into changing conditions. In comparison, recuperability meant the ability to recover from various types of difficulties to re-establish one’s normal state [9, 12].

This scale consists of 20 self-administered items with five items for each dimension. It was scored using a 5-point Likert scale ranging from 1 = strongly disagree to 5 = strongly agree. The total scores ranged from 5–25 for each dimension, and a higher score indicated a higher resilience level [12]. In a previous study, significant results on the t-test (p < 0.001 for all four dimensions) confirmed the instrument’s concurrent validity, and Cronbach Alpha scores (0.76–0.83 for each dimension) confirmed the high internal consistency of the scales [12]. The permission to adapt this questionnaire was kindly given by the author, Robert Taormina of the University of Macau, China.

## Result

### Demographic

The survey was participated by 490 out of 737 registered rural doctors (66% response rate). The completion rate was 100% since all important questions were marked as required. However, after removing outliers, only 454 data were analyzed further. The demographic of these participants are presented in Table 4.

**Table 4:** Participants’ demographic

Variable	N (490)	%	N (454)	%
**Sex**				
Male	202	41.2	185	40.7
Female	288	58.8	269	59.2
**Religion**				
Catholic	208	42.4	170	37.5
Christian	186	38.0	193	42.5
Islam	58	11.8	55	12.1
Hindu	18	3.7	17	3.7
Budha	18	3.7	17	3.7
Kong huchu	1	0.2	1	0.2
N/A	1	0.2	1	0.2
**Age (years)**				
Mean	29.8		29.8	
Median	28.0		27.5	
SD	6.8		7.3	
Minimum	22		23	
Maximum	73		62	

### The psychometric properties

The EFA results showed that the 6-factors model explained the highest cummulative variance of 57.8% (Table 5).

**Table 5:** Exploratory factor analysis (EFA) result

Model	Number of factor	Cummulative variance	RMSEA
1	3 factors	0.455	0.107
2	4 factors	0.503	0.098
3	6 factors	0.578	0.066

The reliability analysis for each factor is presented in Table 6. Cronbach Alpha scores of each factor showed good internal consistencies, which fell between 0.809 and 0.960 (Table 6) [28, 34]. The model fit of the 6-factors model was analyzed using Confirmation Factor Analysis (CFA) and presented in Table 7.

**Table 6:** Dimensions of the 6 factors model

No	Factor	Item	Eigenvalue	Variance		Cronbach α
				(%)	(Cumulative %)	
1	Life calling*	Q38–42	4.055	13.5	13.5	0.960
2	Recuperability	Q16–20	3.263	10.9	24.4	0.918
3	Endurance	Q6–10	3.160	10.5	34.9	0.896
4	Comfort zone*	Q31, 33–34, 36–37	2.298	7.7	42.6	0.823
5	Adaptability	Q11–15	2.575	8.6	51.2	0.868
6	Determination	Q1–5	1.992	6.6	57.8	0.809

^*^Local dimension

**Table 7:** Confirmatory factor analysis (CFA) fit indices of the 6 factors model

Model	CFI	TLI	NNFI	RNI
6 Factors model	0.914	0.904	0.904	0.914

*CFI* Comparative Fit Index; *TLI* Tucker–Lewis Index; *NNFI* Bentler–Bonett Non-normed Fit Index; *RNI* Relative Noncentrality Index

This 6-factors model consisted of the four original dimensions and two additional local dimensions (Table 6). The items’ detail of the local dimensions is presented in Table 8.

**Table 8:** Two factors consisting of local items

Dimension	Q No	Final item
Comfort zone	Q31	This is the right place for me
	Q33	I am financially better off here than in other places
	Q34	Since I started working here, I have been able to achieve many of my goals (dreams)
	Q36	I enjoy living in this place
	Q37	Moving here for work was a good decision
Life calling	Q38	I believe that God has a purpose in placing me here
	Q39	God has always guided me throughout my life
	Q40	God would never allow me to walk through life alone
	Q41	Even in difficult situations, God has been there to guide me
	Q42	I believe that God is ever-present in everything I do

## Discussion

The adapted questionnaire is valid and reliable for measuring resilience in Indonesian rural doctors’ context as this study aims. The psychometric properties of the adapted questionnaire were equivalent to the original questionnaire. The Cronbach alpha of the adapted version was comparable to the original questionnaire, which ranged from 0.81–0.96 and 0.76–0.83 for each dimension, respectively [12].

The experience described here shows that combining several linguistic and cultural adaption methods increased the rigor of the process beyond a single back-translation procedure. The combined methods involve an indigenous approach study [20], forward and backward translations, a committee approach, communication with the original author for meaning equivalence, and pre-testing to bilingual target participants. This study is aligned with previous studies’ suggestions to use a combined approach [18, 19, 35]. However, choosing methods for adaptation is a matter of the researcher’s preference and logistics [19].

There are six dimensions in the adapted questionnaire consist of the four original dimensions and two new additional local dimensions labeled as ‘Comfort zone’ and ‘Life calling.’ The local dimensions resulted from the implemented indigenous psychological approach. This approach explored the local culture and behavior to enrich the questionnaire and better portray and represent the local context in the adapted questionnaire.

The ‘Comfort zone’ dimension was defined as the sense of positive feeling, constructed by habit, which a person has within their current condition. In other words, the person can accept and feel well enough within his current personal condition. The word ‘zone’ means psychological zone. However, it could not be detached from the place where the person lives (physical zone) as the context. The concept of a comfort zone is aligned with the concept of satisfaction with life which has a positive correlation with resiliency [36], and a sense of relatedness to others which is a protective factor of resiliency [37]. Also, a sense of place and individual attachment to a rural area, either physical or psychological, were found to play an essential role in doctor retention [38, 39]. Taking time for outdoor recreation and enjoy nature were described as positive coping strategies toward resilience [5].

The ‘Life calling’ dimension was defined as the sense of personal fulfillment and meaning from believing that what they did had a socially valuable purpose [40] and economic properties cannot substitute. This sense was believed as related to the personal relationship built with the person’s God. This dimension aligned with previous studies identifying spirituality and religion as resilience factors [41]. Resilience was developed through faith in the power of a person’s God [8] which provides life purpose and assists one in finding meaning beyond the adversity experienced [41, 42] through practicing sharing within a faith-based community, prayer, and meditation [42]. This is aligned with the world values survey in which different societies have different perceptions of the importance of religion and how religion influences people’s behavior. In some societies, including Indonesia, religion strongly influences people’s values and daily behavior [43]. However, the cultural differences between societies may limit the generalizability of the findings.

All items in the endurance dimension are written with positive item stems, but the EFA result yielded all negative loadings. The EFA result means that all of the endurance items are in the same direction. The negative loading implies that the factor is located in a different quadrant in the EFA compared to other factors. It does not change the magnitude of the relationship between items or factors [29].

There are several limitations to the study. The generalisability of the findings is limited to communities with similar characteristics to the study participants who were majority Christians, doctors, and living in rural areas. To improve the generalisability of the findings, further studies with more heterogenous representatives, including other health professionals, are needed. However, the results align with the purpose of the study to develop a scale specific for measuring the doctors’ resilience as part of a larger project aiming to develop Indonesian rural doctors’ resilience.

Despite its limitations, this study benefits the community by setting a foundation and providing a tool for future studies on measuring, developing, and evaluating resilience, especially in the Indonesian rural contexts. It also shows the importance of implementing an indigenous approach in developing a questionnaire that fits for purpose and context.

## Conclusion

A personal resilience questionnaire for rural doctors in Indonesia has been successfully developed by incorporating an indigenous approach into the process of cross-cultural adaptation of an existing Four Dimensions Adult Personal Resilience Questionnaire. In such an indigenous approach the focus is not only on translation, but also on transculturation to ensure that the depth and breadt of the pscyhologcial construct is captured comprehensively. The EFA confirms the six-factors solution to have the highest cumulative variance. There are two additional local dimensions (Comfort zone and Life calling) due to the indigenous approach. The final 30 items adapted version of the Adult Personal Resilience Questionnaire that consists of the six dimensions of resilience is valid and reliable for measuring resilience in the Indonesian rural health workers’ context.

## Supplementary Information

**Additional file 1.** Rural doctors' resilience data.

## Data Availability

The dataset supporting the conclusions of this article is included within the article (and its additional file 1).

## Abbreviations

CCA: Cross-cultural adaptation
NTT: Nusa Tenggara Timur
SMS: Short message service
GP: General practitioner
APRQ: Adult Personal Resilience Questionnaire
EFA: Exploratory factor analysis
KMO: Kaiser–Mayer–Olkin
